# Tetra­kis(dihydrogen pefloxacinium) di-μ_2_-chlorido-bis­[tetra­chloridobismuthate(III)] tetra­chloride octa­hydrate

**DOI:** 10.1107/S1600536808017674

**Published:** 2008-06-19

**Authors:** A. V. Polishchuk, A. V. Gerasimenko, K. A. Gayvoronskaya, E. T. Karaseva

**Affiliations:** a Institute of Chemistry, FEB RAS, Prospekt 100-letiya Vladivostoka 159, Vladivostok 690022, Russian Federation

## Abstract

The title compound {systematic name: tetra­kis[4-(3-carb­oxy-1-ethyl-6-fluoro-4-hydroxonio-1,4-dihydro-7-quinol­yl)-1-meth­yl­piperazin-1-ium] di-μ_2_-chlorido-bis­[tetra­chlorido­bismuth­ate(III)] tetra­chloride octa­hydrate}, (C_17_H_22_FN_3_O_3_)_4_[Bi_2_Cl_10_]Cl_4_·8H_2_O, is composed of edge-shared centrosymmetric dinuclear [Bi_2_Cl_10_]^4−^ anions, Cl^−^ anions, dihydrogen pefloxacinium cations and water mol­ecules. The Bi^III^ coordination polyhedron is a distorted octa­hedron. There are four short terminal Bi—Cl bonds [2.5037 (10)–2.6911 (7) Å] and two longer bridging bonds [2.8834 (8) and 3.0687 (9) Å] in each octa­hedron. Two sets of chloride ions and water mol­ecules are disordered over the same sites with site occupancies of 1/3 and 2/3, respectively. Anions, cations and water mol­ecules are linked by O—H⋯O, O—H⋯Cl and N—H⋯Cl hydrogen bonds, forming a three-dimensional framework. There are also π–π stacking inter­actions between quinoline ring systems [centroid–centroid distance = 3.575 (1) Å].

## Related literature

For a description of the Cambridge Structural Database, see: Allen (2002 ▶).
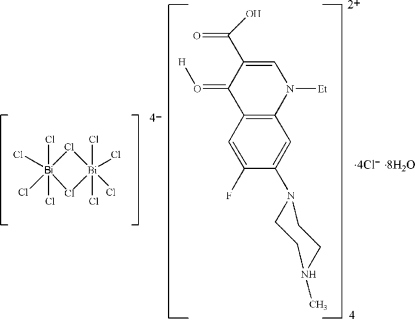


## Experimental

### 

#### Crystal data

(C_17_H_22_FN_3_O_3_)_4_[Bi_2_Cl_10_]Cl_4_·8H_2_O
*M* *_r_* = 2399.89
Monoclinic, 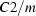

*a* = 14.4201 (14) Å
*b* = 25.305 (3) Å
*c* = 12.6359 (12) Å
β = 99.028 (2)°
*V* = 4553.7 (8) Å^3^
*Z* = 2
Mo *K*α radiation
μ = 4.35 mm^−1^
*T* = 173 (2) K
0.30 × 0.05 × 0.01 mm

#### Data collection

Bruker SMART 1000 CCD area-detector diffractometer
Absorption correction: Gaussian (*XPREP*, *SADABS*; Bruker, 2003 ▶) *T*_min_ = 0.606, *T*_max_ = 0.958
16488 measured reflections
6329 independent reflections
5310 reflections with *I* > 2σ(*I*)
*R*_int_ = 0.048

#### Refinement

*R*[*F* ^2^ > 2σ(*F* ^2^)] = 0.034
*wR*(*F* ^2^) = 0.077
*S* = 1.08
6329 reflections
303 parameters
3 restraints
H atoms treated by a mixture of independent and constrained refinement
Δρ_max_ = 1.23 e Å^−3^
Δρ_min_ = −1.17 e Å^−3^

### 

Data collection: *SMART* (Bruker, 1998 ▶); cell refinement: *SAINT* (Bruker, 2003 ▶); data reduction: *SAINT* ; program(s) used to solve structure: *SHELXTL* (Sheldrick, 2008 ▶); program(s) used to refine structure: *SHELXTL* ; molecular graphics: *XP* in *SHELXTL*; software used to prepare material for publication: *publCIF* (Westrip, 2008 ▶).

## Supplementary Material

Crystal structure: contains datablocks I, global. DOI: 10.1107/S1600536808017674/ci2612sup1.cif

Structure factors: contains datablocks I. DOI: 10.1107/S1600536808017674/ci2612Isup2.hkl

Additional supplementary materials:  crystallographic information; 3D view; checkCIF report

## Figures and Tables

**Table:** 

Bi—Cl2	2.5037 (10)
Bi—Cl4	2.5737 (11)
Bi—Cl3	2.6910 (7)
Bi—Cl3^i^	2.6911 (7)
Bi—Cl1	2.8834 (8)
Bi—Cl1^ii^	3.0687 (9)

**Table:** 

O6⋯O6^i^	2.822 (6)
O6⋯Cl6^iii^	2.863 (5)
O6⋯O5^iv^	2.961 (4)
O6⋯O6^iii^	3.074 (6)
O6⋯O3^v^	3.091 (4)
O7⋯Cl7^vi^	2.888 (7)
O7⋯O7^vi^	3.146 (14)

Symmetry codes: (i) 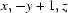; (ii) 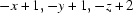; (iii) 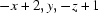; (iv) 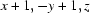; (v) 
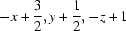
; (vi) 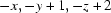.

**Table 2:** Hydrogen-bond geometry (Å, °)

*D*—H⋯*A*	*D*—H	H⋯*A*	*D*⋯*A*	*D*—H⋯*A*
O2—H4⋯Cl5^vii^	0.84	2.18	3.014 (2)	174
O3—H3⋯O1	0.84	1.96	2.675 (3)	143
O3—H3⋯Cl6^viii^	0.84	2.06	2.559 (5)	118
O5—H5*B*⋯O1^ix^	0.83 (1)	2.009 (18)	2.790 (3)	157 (4)
O5—H5*C*⋯O7^x^	0.83 (1)	2.029 (13)	2.851 (5)	173 (3)
O5—H5*C*⋯Cl7^x^	0.83 (1)	2.238 (13)	3.056 (4)	170 (3)
N2—H2⋯Cl1^xi^	0.93	2.52	3.262 (2)	137
N2—H2⋯Cl3^xii^	0.93	2.77	3.423 (2)	128

Symmetry codes: (vii) 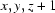; (viii) 
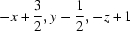
; (ix) 
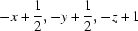
; (x) 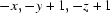; (xi) 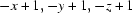; (xii) 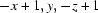.

**Fig. 1.**
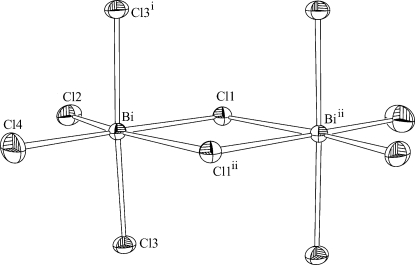
A view of the dinuclear [Bi2F10]4- complex, with displacement ellipsoids drawn at the 50% probability level. [Symmetry codes: x, 1-y, z; (ii) 1-x, 1-y, 2-z.]

**Fig. 2.**
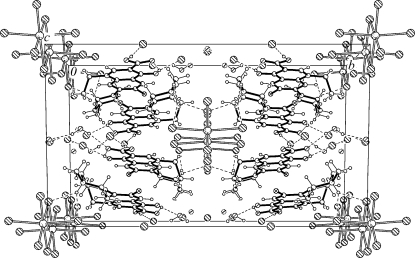
The crystal structure of the title compound, viewed along the c axis. Dashed lines represent hydrogen bonds.

**Fig. 3.**
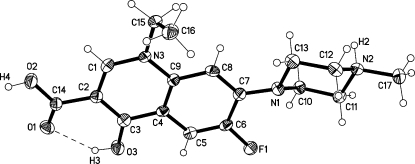
A view of the pfH32+ canion, with displacement ellipsoids drawn at the 50% probability level. The intramolecular hydrogen bond is shown as a dashed line.

**Fig. 4.**
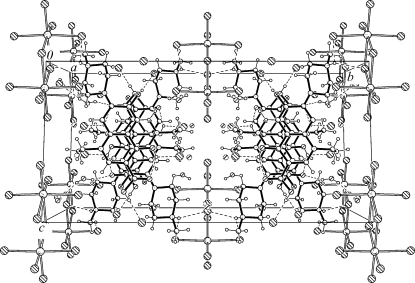
The crystal structure of the title compound, viewed along the a axis. Dashed lines represent hydrogen bonds.

## supplementary crystallographic information

### Comment

Pefloxacin (*pfH*) belongs to the second-generation quinolone antimicrobial agents. According to a search of the Cambridge Structural Database (CSD, Version 5.28; Allen, 2002), well determined structures are those where pefloxacin acts as an anion or as a single protonated cation. The present research deals with the synthesis and structure of a chlorido-bismuth complex with the doubly protonated cation of pefloxacin (*pfH_3_*)^2+^.

The asymmetric unit of the title compound, (I), contains one Bi atom, seven chlorine atoms (two of them are disordered), one *pfH_3_* cation and three H_2_O molecules (from them a two are disordered). The Bi atoms are coordinated by six Cl atoms in a distorted octahedral geometry. Two Bi-centred octahedra are linked by double Cl bridges to form a centrosymmetric dinuclear [Bi_2_F_10_]^4-^ complex (Fig. 1), with a Bi···Bi^i^ distance of 4.4596 (5) Å. In the Bi-octahedra there are four short terminal Bi—Cl bonds [2.5037 (10)-2.6911 (7) Å] and two longer bridging bonds [2.8834 (8) and 3.0687 (9) Å]. These Bi-anions pack up in columns parallel to the [0 0 1] direction (Fig. 2).

The protonation of *pfH_3_*^2+^ is realised on the carbonyl atom O3 and N2 of the piperazine ring (Fig. 3). The hydrogen atom H3 is linked by an intramolecular hydrogen bond with O1 atom of the carboxyl group. Atoms O2 and N2 in the cation act as hydrogen-bond donors, *via* H4 and H2

There are three uncoordinated chlorine atoms (Cl5, Cl6 and Cl7) of which Cl6 and Cl7 are disordered and statistically replaced by atoms O6 and O7 of water molecules, respectively [Wyckoff positions 8j and 4i for Cl6/O6 and Cl7/O7, respectively]. Site occupation factors of these chloride ions were assigned equal to 1/3, and water molecule to 2/3 from the crystal chemistry considerations. The refinement of the Cl6/O6 and Cl7/O7 site occupation factors resulted in the same values with accuracy within 0.04. As the hydrogen atoms were not located for disordered water molecules, probable hydrogen bonds involving these atoms are given in Table 3.

In the crystal structure, the cations are packed along the *a*axis. There exist π–π stacking interactions between quinoline ring systems (Fig.4), with nearest C···C contacts are in the range 3.292 (5)-3.365 (3) Å. Anions, cations and H_2_O-molecules are linked by a network of O—H···O, O—H···Cl and N—H···Cl hydrogen bonds into a three-dimensional framework.

### Experimental

Bi(OH)_3_ (1.00 g, 3.85 mmol) was reacted with *pfH* (1.50 g, 5.77 mmol) in a solution of HCl (35%, 15 ml). Yellow crystals were obtained after evaporation for 72 h at room temperature.

### Refinement

H atoms of H_2_O were located in a difference map and refined with *U*_iso_(H) = 1.5*U*_eq_(O) and the O-H distances were restrained to be similar. The other H atoms were positioned with idealized geometry using a riding model with C-H = 0.95, 0.98 and 0.99 Å; N-H = 0.93 Å and O-H = 0.84 Å. All H atoms were refined with U_iso_ set to 1.2 or 1.5 times *U*_eq_ of the parent atom. Atoms Cl6 and O6, and also Cl7 and O7, are disordered between them with site occupancies of 1/3 and 2/3, respectively. H atoms belonging to the disordered water molecules could not be located. The maximum peak and the deepest hole are located 0.86 Å and 1.13 Å from Bi, respectively.

### Crystal data

**Table:**

(C_17_H_22_FN_3_O_3_)_4_[Bi_2_Cl_10_]Cl_4_·8H_2_O	*F*_000_ = 2392
*M**_r_* = 2399.89	*D*_x_ = 1.750 Mg m^−^^3^
Monoclinic, *C*2/*m*	Mo *K*α radiation λ = 0.71073 Å
Hall symbol: -C 2y	Cell parameters from 897 reflections
*a* = 14.4201 (14) Å	θ = 2.8–27.5º
*b* = 25.305 (3) Å	µ = 4.35 mm^−^^1^
*c* = 12.6359 (12) Å	*T* = 173 (2) K
β = 99.028 (2)º	Prism, yellow
*V* = 4553.7 (8) Å^3^	0.30 × 0.05 × 0.01 mm
*Z* = 2	

### Data collection

**Table:**

Bruker SMART 1000 CCD area-detector diffractometer	6329 independent reflections
Radiation source: fine-focus sealed tube	5310 reflections with *I* > 2σ(*I*)
Monochromator: graphite	*R*_int_ = 0.048
Detector resolution: 8.33 pixels mm^-1^	θ_max_ = 29.5º
*T* = 173(2) K	θ_min_ = 1.6º
ω scans	*h* = −16→19
Absorption correction: Gaussian(SADABS; Bruker, 2003)	*k* = −34→30
*T*_min_ = 0.606, *T*_max_ = 0.958	*l* = −17→8
16488 measured reflections	

### Refinement

**Table:**

Refinement on *F*^2^	Secondary atom site location: difference Fourier map
Least-squares matrix: full	Hydrogen site location: inferred from neighbouring sites
*R*[*F*^2^ > 2σ(*F*^2^)] = 0.034	H atoms treated by a mixture of independent and constrained refinement
*wR*(*F*^2^) = 0.077	*w* = 1/[σ^2^(*F*_o_^2^) + (0.0241*P*)^2^ + 5.6647*P*] where *P* = (*F*_o_^2^ + 2*F*_c_^2^)/3
*S* = 1.08	(Δ/σ)_max_ = 0.010
6329 reflections	Δρ_max_ = 1.23 e Å^−^^3^
303 parameters	Δρ_min_ = −1.17 e Å^−^^3^
3 restraints	Extinction correction: none
Primary atom site location: structure-invariant direct methods	

### Special details

**Table:**

Geometry. All e.s.d.'s (except the e.s.d. in the dihedral angle between two l.s. planes) are estimated using the full covariance matrix. The cell e.s.d.'s are taken into account individually in the estimation of e.s.d.'s in distances, angles and torsion angles; correlations between e.s.d.'s in cell parameters are only used when they are defined by crystal symmetry. An approximate (isotropic) treatment of cell e.s.d.'s is used for estimating e.s.d.'s involving l.s. planes.
Refinement. Refinement of *F*^2^ against ALL reflections. The weighted *R*-factor *wR* and goodness of fit *S* are based on *F*^2^, conventional *R*-factors *R* are based on *F*, with *F* set to zero for negative *F*^2^. The threshold expression of *F*^2^ > σ(*F*^2^) is used only for calculating *R*-factors(gt) *etc*. and is not relevant to the choice of reflections for refinement. *R*-factors based on *F*^2^ are statistically about twice as large as those based on *F*, and *R*- factors based on ALL data will be even larger.

### Fractional atomic coordinates and isotropic or equivalent isotropic displacement parameters (Å^2^)

**Table:**

	*x*	*y*	*z*	*U*_iso_*/*U*_eq_	Occ. (<1)
Bi	0.478467 (9)	0.5000	1.170934 (10)	0.01789 (3)	
Cl1	0.36184 (6)	0.5000	0.96352 (7)	0.02166 (18)	
Cl2	0.32976 (7)	0.5000	1.24998 (8)	0.0332 (2)	
Cl3	0.46439 (4)	0.39397 (2)	1.16382 (5)	0.02578 (14)	
Cl4	0.58921 (10)	0.5000	1.35173 (9)	0.0501 (3)	
Cl5	0.5000	0.19810 (4)	0.0000	0.0277 (2)	
Cl6	0.9645 (3)	0.58510 (18)	0.4025 (3)	0.0829 (12)	0.33333
O6	0.9296 (2)	0.55576 (12)	0.3952 (3)	0.0376 (8)	0.66667
Cl7	0.0257 (3)	0.5000	0.9040 (4)	0.0483 (12)	0.33333
O7	0.0152 (4)	0.5000	0.8794 (5)	0.0447 (17)	0.66667
F2	0.69789 (11)	0.22163 (6)	0.19969 (11)	0.0286 (4)	
O1	0.55734 (13)	0.17802 (7)	0.73845 (15)	0.0286 (5)	
O2	0.56440 (14)	0.25726 (7)	0.81762 (14)	0.0302 (5)	
H4	0.5504	0.2396	0.8692	0.045*	
O3	0.60949 (13)	0.17118 (7)	0.54491 (15)	0.0283 (5)	
H3	0.5969	0.1586	0.6025	0.042*	
O5	0.05523 (17)	0.41020 (10)	0.2439 (2)	0.0570 (7)	
H5B	0.0279 (12)	0.3815 (5)	0.234 (3)	0.085*	
H5C	0.0302 (18)	0.4357 (6)	0.210 (2)	0.085*	
N1	0.70541 (15)	0.33043 (8)	0.19082 (16)	0.0218 (5)	
N2	0.74434 (15)	0.38831 (9)	0.00739 (17)	0.0235 (5)	
H2	0.6895	0.4083	−0.0053	0.028*	
N3	0.62148 (14)	0.33321 (8)	0.55077 (16)	0.0198 (5)	
C1	0.60166 (16)	0.30659 (10)	0.6364 (2)	0.0215 (6)	
H1A	0.5905	0.3263	0.6972	0.026*	
C2	0.59658 (16)	0.25233 (10)	0.6410 (2)	0.0203 (6)	
C3	0.61226 (17)	0.22153 (11)	0.5512 (2)	0.0227 (6)	
C4	0.63379 (16)	0.25024 (10)	0.45924 (19)	0.0187 (5)	
C5	0.65146 (16)	0.22261 (10)	0.36745 (19)	0.0209 (6)	
H5	0.6481	0.1851	0.3656	0.025*	
C6	0.67306 (17)	0.24941 (10)	0.28238 (19)	0.0212 (6)	
C7	0.67653 (16)	0.30550 (10)	0.27776 (19)	0.0206 (6)	
C8	0.65728 (17)	0.33298 (10)	0.36801 (19)	0.0209 (6)	
H8	0.6572	0.3705	0.3677	0.025*	
C9	0.63802 (16)	0.30572 (10)	0.45928 (19)	0.0191 (5)	
C10	0.65575 (17)	0.31800 (11)	0.08222 (19)	0.0220 (6)	
H10A	0.5970	0.3388	0.0675	0.026*	
H10B	0.6391	0.2800	0.0778	0.026*	
C11	0.71759 (18)	0.33095 (11)	0.0000 (2)	0.0249 (6)	
H11A	0.7749	0.3088	0.0126	0.030*	
H11B	0.6838	0.3231	−0.0726	0.030*	
C12	0.79311 (18)	0.40046 (11)	0.1187 (2)	0.0253 (6)	
H12A	0.8084	0.4386	0.1245	0.030*	
H12B	0.8527	0.3804	0.1333	0.030*	
C13	0.73148 (19)	0.38594 (11)	0.2011 (2)	0.0250 (6)	
H13A	0.7657	0.3928	0.2740	0.030*	
H13B	0.6742	0.4081	0.1904	0.030*	
C14	0.57179 (17)	0.22525 (10)	0.73660 (19)	0.0214 (6)	
C15	0.62925 (18)	0.39181 (10)	0.5556 (2)	0.0248 (6)	
H15A	0.5993	0.4049	0.6159	0.030*	
H15B	0.5948	0.4070	0.4886	0.030*	
C16	0.72972 (19)	0.41048 (11)	0.5703 (2)	0.0293 (7)	
H16A	0.7600	0.4031	0.6438	0.044*	
H16B	0.7312	0.4486	0.5569	0.044*	
H16C	0.7633	0.3919	0.5198	0.044*	
C17	0.8038 (2)	0.40306 (12)	−0.0740 (2)	0.0336 (7)	
H17A	0.8594	0.3802	−0.0661	0.050*	
H17B	0.8235	0.4400	−0.0635	0.050*	
H17C	0.7678	0.3987	−0.1459	0.050*	

### Atomic displacement parameters (Å^2^)

**Table:**

	*U*^11^	*U*^22^	*U*^33^	*U*^12^	*U*^13^	*U*^23^
Bi	0.02397 (6)	0.01417 (5)	0.01620 (5)	0.000	0.00521 (4)	0.000
Cl1	0.0236 (4)	0.0201 (4)	0.0211 (4)	0.000	0.0032 (3)	0.000
Cl2	0.0413 (4)	0.0254 (4)	0.0391 (5)	0.000	0.0257 (4)	0.000
Cl3	0.0324 (3)	0.0162 (3)	0.0304 (3)	0.0004 (2)	0.0101 (3)	0.0003 (2)
Cl4	0.0714 (8)	0.0404 (6)	0.0299 (5)	0.000	−0.0188 (5)	0.000
Cl5	0.0309 (4)	0.0253 (4)	0.0282 (4)	0.000	0.0085 (4)	0.000
Cl6	0.079 (2)	0.088 (3)	0.084 (2)	−0.014 (2)	0.0203 (19)	−0.022 (2)
O6	0.058 (2)	0.0163 (14)	0.0394 (17)	0.0067 (14)	0.0099 (15)	0.0009 (13)
Cl7	0.050 (2)	0.0267 (17)	0.058 (2)	0.000	−0.0206 (18)	0.000
O7	0.042 (3)	0.038 (3)	0.050 (3)	0.000	−0.004 (3)	0.000
F2	0.0418 (8)	0.0238 (8)	0.0208 (7)	0.0034 (7)	0.0070 (6)	−0.0058 (6)
O1	0.0358 (10)	0.0205 (9)	0.0294 (10)	0.0011 (8)	0.0042 (8)	0.0024 (7)
O2	0.0472 (10)	0.0221 (9)	0.0239 (9)	−0.0025 (8)	0.0138 (8)	−0.0013 (7)
O3	0.0393 (10)	0.0200 (9)	0.0267 (9)	−0.0044 (8)	0.0087 (8)	0.0007 (7)
O5	0.0548 (14)	0.0491 (15)	0.0700 (17)	−0.0072 (12)	0.0190 (13)	0.0058 (13)
N1	0.0286 (10)	0.0186 (10)	0.0177 (10)	−0.0028 (9)	0.0020 (8)	−0.0021 (8)
N2	0.0248 (10)	0.0225 (11)	0.0235 (10)	0.0054 (9)	0.0047 (8)	0.0037 (8)
N3	0.0231 (9)	0.0181 (10)	0.0188 (9)	0.0037 (8)	0.0052 (8)	−0.0024 (8)
C1	0.0185 (10)	0.0255 (13)	0.0210 (11)	−0.0011 (10)	0.0048 (9)	−0.0041 (10)
C2	0.0162 (10)	0.0235 (12)	0.0212 (11)	−0.0002 (9)	0.0026 (9)	−0.0001 (9)
C3	0.0188 (11)	0.0258 (13)	0.0226 (12)	−0.0009 (10)	0.0005 (10)	−0.0026 (10)
C4	0.0143 (10)	0.0214 (12)	0.0197 (11)	0.0022 (9)	0.0004 (9)	−0.0020 (9)
C5	0.0188 (11)	0.0200 (12)	0.0233 (12)	−0.0019 (9)	0.0016 (9)	−0.0038 (9)
C6	0.0203 (11)	0.0239 (12)	0.0190 (11)	0.0043 (10)	0.0021 (9)	−0.0060 (9)
C7	0.0173 (10)	0.0238 (12)	0.0198 (11)	0.0006 (9)	0.0007 (9)	−0.0009 (9)
C8	0.0233 (11)	0.0156 (11)	0.0223 (12)	0.0027 (9)	−0.0010 (10)	−0.0024 (9)
C9	0.0180 (10)	0.0224 (12)	0.0159 (11)	0.0007 (9)	0.0001 (9)	−0.0029 (9)
C10	0.0236 (11)	0.0260 (13)	0.0157 (11)	0.0008 (10)	0.0009 (9)	−0.0016 (9)
C11	0.0291 (12)	0.0244 (13)	0.0221 (12)	0.0028 (11)	0.0062 (10)	−0.0022 (10)
C12	0.0259 (12)	0.0226 (13)	0.0264 (13)	−0.0001 (10)	0.0012 (10)	0.0034 (10)
C13	0.0306 (13)	0.0228 (13)	0.0204 (12)	−0.0016 (11)	0.0006 (10)	−0.0047 (10)
C14	0.0184 (11)	0.0261 (13)	0.0199 (11)	0.0009 (10)	0.0030 (9)	0.0011 (10)
C15	0.0318 (12)	0.0199 (12)	0.0238 (12)	0.0043 (10)	0.0078 (10)	−0.0025 (10)
C16	0.0367 (14)	0.0227 (13)	0.0297 (13)	−0.0025 (11)	0.0085 (11)	−0.0062 (11)
C17	0.0360 (14)	0.0361 (16)	0.0314 (14)	0.0074 (12)	0.0140 (12)	0.0112 (12)

### Geometric parameters (Å, °)

**Table:**

Bi—Cl2	2.5037 (10)	C2—C14	1.480 (4)
Bi—Cl4	2.5737 (11)	C3—C4	1.445 (4)
Bi—Cl3	2.6910 (7)	C4—C9	1.405 (3)
Bi—Cl3^i^	2.6911 (7)	C4—C5	1.411 (3)
Bi—Cl1	2.8834 (8)	C5—C6	1.348 (4)
Bi—Cl1^ii^	3.0687 (9)	C5—H5	0.95
Bi—Bi^ii^	4.4596 (5)	C6—C7	1.422 (4)
Cl1—Bi^ii^	3.0687 (9)	C7—C8	1.400 (4)
F2—C6	1.353 (3)	C8—C9	1.408 (4)
O1—C14	1.214 (3)	C8—H8	0.95
O2—C14	1.323 (3)	C10—C11	1.507 (4)
O2—H4	0.84	C10—H10A	0.99
O3—C3	1.277 (3)	C10—H10B	0.99
O3—H3	0.84	C11—H11A	0.99
O5—H5B	0.828 (10)	C11—H11B	0.99
O5—H5C	0.826 (11)	C12—C13	1.516 (4)
N1—C7	1.386 (3)	C12—H12A	0.99
N1—C13	1.455 (3)	C12—H12B	0.99
N1—C10	1.479 (3)	C13—H13A	0.99
N2—C17	1.486 (4)	C13—H13B	0.99
N2—C11	1.501 (3)	C15—C16	1.507 (4)
N2—C12	1.503 (3)	C15—H15A	0.99
N2—H2	0.93	C15—H15B	0.99
N3—C1	1.343 (3)	C16—H16A	0.98
N3—C9	1.401 (3)	C16—H16B	0.98
N3—C15	1.488 (3)	C16—H16C	0.98
C1—C2	1.377 (4)	C17—H17A	0.98
C1—H1A	0.95	C17—H17B	0.98
C2—C3	1.424 (4)	C17—H17C	0.98
			
O6···O6^i^	2.822 (6)	O6···O3^v^	3.091 (4)
O6···Cl6^iii^	2.863 (5)	O7···Cl7^vi^	2.888 (7)
O6···O5^iv^	2.961 (4)	O7···O7^vi^	3.146 (14)
O6···O6^iii^	3.074 (6)		
			
Cl2—Bi—Cl4	95.56 (4)	N1—C7—C8	122.8 (2)
Cl2—Bi—Cl3	87.046 (14)	N1—C7—C6	120.2 (2)
Cl4—Bi—Cl3	93.594 (14)	C8—C7—C6	116.7 (2)
Cl2—Bi—Cl3^i^	87.046 (14)	C7—C8—C9	120.9 (2)
Cl4—Bi—Cl3^i^	93.594 (14)	C7—C8—H8	119.6
Cl3—Bi—Cl3^i^	171.10 (3)	C9—C8—H8	119.6
Cl2—Bi—Cl1	87.06 (3)	N3—C9—C4	118.9 (2)
Cl4—Bi—Cl1	177.38 (4)	N3—C9—C8	120.9 (2)
Cl3—Bi—Cl1	86.529 (14)	C4—C9—C8	120.2 (2)
Cl3^i^—Bi—Cl1	86.529 (14)	N1—C10—C11	109.9 (2)
Cl2—Bi—Cl1^ii^	170.06 (3)	N1—C10—H10A	109.7
Cl4—Bi—Cl1^ii^	94.38 (4)	C11—C10—H10A	109.7
Cl3—Bi—Cl1^ii^	92.335 (14)	N1—C10—H10B	109.7
Cl3^i^—Bi—Cl1^ii^	92.335 (14)	C11—C10—H10B	109.7
Cl1—Bi—Cl1^ii^	83.00 (3)	H10A—C10—H10B	108.2
Cl2—Bi—Bi^ii^	130.14 (2)	N2—C11—C10	110.0 (2)
Cl4—Bi—Bi^ii^	134.31 (3)	N2—C11—H11A	109.7
Cl3—Bi—Bi^ii^	89.364 (14)	C10—C11—H11A	109.7
Cl3^i^—Bi—Bi^ii^	89.364 (14)	N2—C11—H11B	109.7
Cl1—Bi—Bi^ii^	43.076 (18)	C10—C11—H11B	109.7
Cl1^ii^—Bi—Bi^ii^	39.921 (15)	H11A—C11—H11B	108.2
Bi—Cl1—Bi^ii^	97.00 (2)	N2—C12—C13	110.8 (2)
C14—O2—H4	109.5	N2—C12—H12A	109.5
C3—O3—H3	109.5	C13—C12—H12A	109.5
H5B—O5—H5C	117 (3)	N2—C12—H12B	109.5
C7—N1—C13	118.3 (2)	C13—C12—H12B	109.5
C7—N1—C10	118.5 (2)	H12A—C12—H12B	108.1
C13—N1—C10	111.7 (2)	N1—C13—C12	110.1 (2)
C17—N2—C11	111.8 (2)	N1—C13—H13A	109.6
C17—N2—C12	111.1 (2)	C12—C13—H13A	109.6
C11—N2—C12	109.50 (19)	N1—C13—H13B	109.6
C17—N2—H2	108.1	C12—C13—H13B	109.6
C11—N2—H2	108.1	H13A—C13—H13B	108.2
C12—N2—H2	108.1	O1—C14—O2	123.6 (2)
C1—N3—C9	120.1 (2)	O1—C14—C2	122.4 (2)
C1—N3—C15	119.4 (2)	O2—C14—C2	114.0 (2)
C9—N3—C15	120.5 (2)	N3—C15—C16	112.5 (2)
N3—C1—C2	123.6 (2)	N3—C15—H15A	109.1
N3—C1—H1A	118.2	C16—C15—H15A	109.1
C2—C1—H1A	118.2	N3—C15—H15B	109.1
C1—C2—C3	119.7 (2)	C16—C15—H15B	109.1
C1—C2—C14	121.2 (2)	H15A—C15—H15B	107.8
C3—C2—C14	119.1 (2)	C15—C16—H16A	109.5
O3—C3—C2	126.0 (2)	C15—C16—H16B	109.5
O3—C3—C4	117.4 (2)	H16A—C16—H16B	109.5
C2—C3—C4	116.5 (2)	C15—C16—H16C	109.5
C9—C4—C5	118.8 (2)	H16A—C16—H16C	109.5
C9—C4—C3	121.1 (2)	H16B—C16—H16C	109.5
C5—C4—C3	120.1 (2)	N2—C17—H17A	109.5
C6—C5—C4	120.0 (2)	N2—C17—H17B	109.5
C6—C5—H5	120.0	H17A—C17—H17B	109.5
C4—C5—H5	120.0	N2—C17—H17C	109.5
C5—C6—F2	118.5 (2)	H17A—C17—H17C	109.5
C5—C6—C7	123.3 (2)	H17B—C17—H17C	109.5
F2—C6—C7	118.1 (2)		

Symmetry codes: (i) *x*, −*y*+1, *z*; (ii) −*x*+1, −*y*+1, −*z*+2; (iii) −*x*+2, *y*, −*z*+1; (iv) *x*+1, −*y*+1, *z*; (v) −*x*+3/2, *y*+1/2, −*z*+1; (vi) −*x*, −*y*+1, −*z*+2.

### Hydrogen-bond geometry (Å, °)

**Table:**

*D*—H···*A*	*D*—H	H···*A*	*D*···*A*	*D*—H···*A*
O2—H4···Cl5^vii^	0.84	2.18	3.014 (2)	174
O3—H3···O1	0.84	1.96	2.675 (3)	143
O3—H3···Cl6^viii^	0.84	2.06	2.559 (5)	118
O5—H5B···O1^ix^	0.83 (1)	2.009 (18)	2.790 (3)	157 (4)
O5—H5C···O7^x^	0.83 (1)	2.029 (13)	2.851 (5)	173 (3)
O5—H5C···Cl7^x^	0.83 (1)	2.238 (13)	3.056 (4)	170 (3)
N2—H2···Cl1^xi^	0.93	2.52	3.262 (2)	137
N2—H2···Cl3^xii^	0.93	2.77	3.423 (2)	128

Symmetry codes: (vii) *x*, *y*, *z*+1; (viii) −*x*+3/2, *y*−1/2, −*z*+1; (ix) −*x*+1/2, −*y*+1/2, −*z*+1; (x) −*x*, −*y*+1, −*z*+1; (xi) −*x*+1, −*y*+1, −*z*+1; (xii) −*x*+1, *y*, −*z*+1.
